# Exploring global retailers' corporate social responsibility performance

**DOI:** 10.1016/j.heliyon.2020.e04644

**Published:** 2020-08-13

**Authors:** Amir Rahdari, Benedict Sheehy, Habib Zaman Khan, Udo Braendle, Gadaf Rexhepi, Sahar Sepasi

**Affiliations:** a Sustainability Research Group (SRG), Universal Scientific Education and Research Network (USERN), Tehran, Iran; b Tarbiat Modares University, Tehran, Iran; c Canberra Law School, University of Canberra, ACT, Australia; d Canberra Business School, University of Canberra, ACT, Australia; e Department of General Business and Management, School of Business Administration, American University in Dubai, United Arab Emirates; f South East European University, Macedonia; g Max van der Stoel Institute, South East European University, Macedonia; h Sustainability Research Group (SRG), Universal Scientific Education and Research Network (USERN), Skopje, Macedonia

**Keywords:** Corporate social responsibility, Retail, Strategy, Sustainability, Supply chain, SDG, Sustainable development, Technology management, Entrepreneurship, Business policy, Management, Globalization, Industrialization

## Abstract

Retailers serve as the main interface between business and society. This study explores the Corporate Social Responsibility priorities and performance of the largest 23 global retailers. This set of global retailers, who have a major impact on society, were studied in terms of social, environmental and sustainability practices and strategy, and there performance was analysed and evaluated. The study uses a four-dimensional Social, Economic, Environmental, Supply Chain model for sustainability performance evaluation. We rely on data collected from annual reports, and find that global retailers have addressed the business-society interface in relatively balanced ways for the different dimensions of CSR. Further, our findings indicate that global retailers in different regions have different CSR priorities. In particular, the data indicates that the US retailers place a lower priority on supply chain sustainability performance, followed by the Australians, while European retailers place a higher priority. The study concludes that while global retailers all pay attention to the same dimensions of CSR and do so differently in the different regions, the variation and lack of significant progress indicates that there is a role for stronger government regulation. This study contributes to the literature by shifting the analysis from country to a global level, is more objective in relying on reported data rather than interviews or surveys and provides a new analytical tool.

## 1 Introduction

The retail industry provides the interface for business with society broadly and stands at the apex of the supply chain. In this position it is simultaneously the driver of manufacturing and the face of production to billions of consumers around the globe. In this critical position, its role in driving the global sustainability agenda is critical. Retail is a diverse and dynamic global industry offering a wide range of goods and services to consumers (Erol et al., 2009) with sales of the top 250 global retailers reaching 4.3 trillion dollars in 2015 (Deloitte Touche Tohmatsu, 2017). The industry employs 15.9 million people in the U.S. alone and form about 10% of the total employments (U.S. Bureau of Labor Statistics, 2018). Nearly two-thirds of the U.S. gross domestic product comes from retail industry and it is approximately the same for most countries. Accordingly, understanding what the retail industry is doing is critical to global policy, governance and sustainability generally. Further, understanding the industry's response to stakeholder pressure provides insights not only into stakeholder concerns but also the adaptability and agility of these global giants in response to such pressures and the potential for social pressure to affect their policies and practices.

Although the retail industry comprises a considerable portion of the global economy, it is a particularly vulnerable industry. Because its business model is so broadly embedded within society, both in terms of suppliers in its upstream value chains and social context, and downstream with consumers (Schrempf-Stirling and Palazzo, 2016), it has a significant responsibility towards society. As it relies on stakeholder attitudes, namely consumers' attitudes, it is vulnerable to changes in sentiment based not on the vicissitudes of economics that affect all industries, but on reputation. Retailers fortunes may rise and fall regardless of the objective value of products or services and so must invest to protect their reputations as is evident in the heavy spending on branding and good will generally. Retailers are additionally exposed to vulnerability because they rely on lengthy and extended supply chains often beyond their control. Retailers are not only expected by societal stakeholders to behave responsibly in terms of their own business practices but also to provide products produced sustainably by suppliers. As such they are expected to pressure their suppliers to operate in socially responsible ways—to address issues like unfair labor practices and safety conditions in factories, air pollution from manufacturing, chemical discharge into waterways, deforestation for food production, and water stress in areas where cotton is grown (Vozza, 2017). As a result, many retail retailers in many developed countries concentrate most of their CSR initiatives—initiatives ameliorating conflict at the business-society interface--on supply chain actors.

Previous studies have examined limited aspects of these problems with inadequate data and tools. Previous studies relied on data drawn from interviews and surveys, and while their analytical tools appropriate for the qualitative data, they were unable to move beyond. Accordingly, the current study takes a fresh approach to examining and exploring the corporate social responsibility (CSR) priorities and performance of the 23 largest global retailers and the performance of their supply chains across 3 regions of the globe. It focuses on identifying and evaluating the economic, social, environmental performance, and supply chain performance of these actors. It does so using a new analytical tool, a Social, Economic Environmental, Supply Chain (‘SEES’) model.

The rest of the article is organized in the following manner. The next section provides a brief review of the literature encompassing the theoretical foundations of sustainability, CSR and analytical models with a focus on the retail industry. In the third and fourth sections, the study focuses on method, results and discussions. This study paves the way for future research by examining the state of CSR and sustainability strategy in the retail industry, and challenges for the business-society interface.

## 2 Theoretical background

The issues and terms surrounding the CSR research are broad and complex. Sustainability is a ubiquitous term and means anything from saving the planetary natural environment to making profits for the foreseeable future as a result of a competitive position (Porter, 2008). Sustainability is connected to a global policy discussion on sustainable development (Drexhage and Murphy, 2010). CSR is used interchangeably with a number of terms including sustainability and corporate sustainability among others (Sheehy and Farneti, 2019).

CSR, unlike the broader sustainability agenda or the narrower environmentally focused ‘corporate sustainability’ agenda (Sheehy and Farneti, 2019), is a long standing socio-political movement aimed solely but broadly at businesses with the objective of reducing the social costs associated with industrial activity (Sheehy, 2017). Understood this way, CSR can be defined as a type of emerging business law, a type of private international law or self-regulation (Sheehy, 2015; Ruggie, 2017). It has a regulatory focus in that it aims to create constraints upon and incentives within the business environment to decrease certain harms and promote certain goods (Sheehy and Feaver, 2015).

CSR norms have been used to pressure business informally to address matters from fairness in wages, to environmental impacts, to supply chain issues in addition to formal pressures resulting from direct legislation on these and similar matters. As emerging business law, CSR imposes at a minimum an obligation to report on non-financial performance in terms of social and environmental impacts (KPMG, 2013; Sheehy, 2015). CSR itself is moving increasingly into hard law in various ways and realms (Mares, 2010); however, it is doing so slowly and as a widely divergent phenomenon (Matten and Moon, 2008; Afsharipour and Rana, 2013; Sheehy and Feaver, 2014; Sheehy, 2017). It is important to note that CSR is about addressing responsibility for impacts of operations and not about covering by publicly promulgated laws only. In other words, the binary voluntary-mandatory is as misleading as it is unhelpful in determining whether an activity is addressed by CSR or not. Whether the impacts of a business are covered by law does not indicate whether or not it is a responsibility. Rather, CSR is the responsibility of the business to address regardless of the nature of public authoritative regulation (Mares, 2010).

The current study adopts the managerial branch of stakeholders' theory—i.e. a focus on powerful stakeholders as opposed to the ethical branch which focuses on fairness and justice—in order to illuminate the initiatives taken by global firms in relation to supply chain sustainability. Proponents of stakeholder theory generally, have suggested that there should be a fit between the “values of the corporation and its managers, the expectations of stakeholders and the societal issues which will determine the ability of the firm to sell its products” (Freeman, 2004, p. 5). Stakeholder theory attempts to articulate and broaden the business-society interface beyond the mere commercial.

Based on above-discussion, the current study understands CSR as a moblisation of global leading 23 retailers to improve CSR practices in their supply chain which is a management response to stakeholder pressures.

### 2.1 CSR, sustainability reporting and sustainable supply chains

CSR in the retail sector takes on a different form than in other industries. While other industries within supply chains may address their own immediate environmental, community issues or workplace issues, the focus of much CSR in the retail industry is not a response to its own immediate practices, but from practices in its supply chain and in response to consumer pressure.

This phenomenon of retail pressure being applied in order to change supply chain processes may be the simple result of the distribution of jobs. An estimated one in every seven jobs are supply-chain related. Additionally, the focus on retails' supply chain management is a consequence of product and process standards being set by lead companies such as retailers the results of which cascade down through the supply chain and across the business network (Ruggie, 2017). Research suggests that there is growing consumer and general social pressure on retailers to use their power vis a vis the suppliers as a lever for positive change (Vandenbergh, 2007).

There is a very considerable literature on CSR in non-retail industries, both theoretical and applied. Applied studies have been conducted in both developed and emerging economies and have enhanced our understanding of the influence regulators, media and other local stakeholders exert on firms' to adopt or modify CSR practices (Khan et al., 2014, 2019; Campbell and Slack, 2011; Cooper and Owen, 2007; Jamali and Karam, 2018). In the specific context of retail industries, recent studies have advanced our understanding of the power and pressure that global retailers have and exert on their suppliers to engage in CSR practices (Islam and Deegan, 2010). On the related advancement of CSR in the retail industry generally, one line of research has focused on sustainability in supply chains. For example, scholars have examined drivers of sustainable supply chain performance (Zhu and Sarkis, 2006), provided an overview of issues essential for environmental performance in sustainable supply chain, developed a strategic decision framework for sustainable supply chain management (Sarkis et al., 2011), CSR reporting and its relationship with corporate governance elements (Khan, 2010) and reviewed sustainability reporting practices using the GRI framework (Khan et al., 2011).

The sustainable supply chain literature is still new but indicates that researchers have taken several focal points and approaches to their studies. For example, they have investigated how managers have attempted to integrate sustainability into their business models. They have developed different initiatives for sustainable logistic management and have documented the complexity associated with sustainable supply chain practices (Amini and Bienstock, 2014). The earlier work of Formentini and Taticchi identified the state of the art and set out a research agenda, nominating for special attention, the role of governance mechanisms for promoting sustainable supply chain management (Formentini and Taticchi, 2016). Conceptual work developing a framework for sustainable supply chain management analysis provided a way forward in the research (Seuring and Muller, 2008), while Walker and Jones focused their research on understanding internal and external barriers, and internal and external enablers of sustainable supply chain management in the retail industry (Walker and Jones, 2012).

A distinct line of research studies focuses on sustainable supply chain management practice and its linkage to organizational performance (Seuring and Gold, 2013). The literature in this field has been inconclusive. For example, while some studies have found a direct and positive relationship between sustainable supply chain management practices and organizational performance (Zhu et al., 2005), other studies have reported a negative relationship. Yet other studies have drawn attention to the necessity of considering moderating and mediating variables in understanding the relationships (Zhu et al., 2012). One significant weakness of many of these studies is their reliance on surveys to examine managerial perceptions of organizational performance instead of more concrete and objective organizational performance data. While such data are available from publicly available sources such as firms' website disclosures relating to sustainability reporting, CSR reports, more general annual reports (e.g. Zhu et al., 2012), the prior research does not appear to engage with it as much is it could.

A final limitation in the prior research is the issue of focus. Previous studies have of sustainable supply chain performance have been focused on the national level, for example, in the US (Wang and Sarkis, 2013), UK (Walker and Jones, 2012) or in emerging economies such as China (Zhu et al., 2005). The current study aims to expand on our understanding of these issues by taking focusing on global firms operating across a wide variety of nations and doing so using empirical data.

### 2.2 Conceptual framework

Overall, the study has following RQs.

**(RQ 1) What objectively are the sustainability priorities and CSR performance of the largest global retailers?**

**(RQ 2) How does the CSR performance of global retailers compare objectively across three regional markets?**

#### 2.2.1 The Retail Industry Leaders Association performance evaluation model

**Figure 1:**
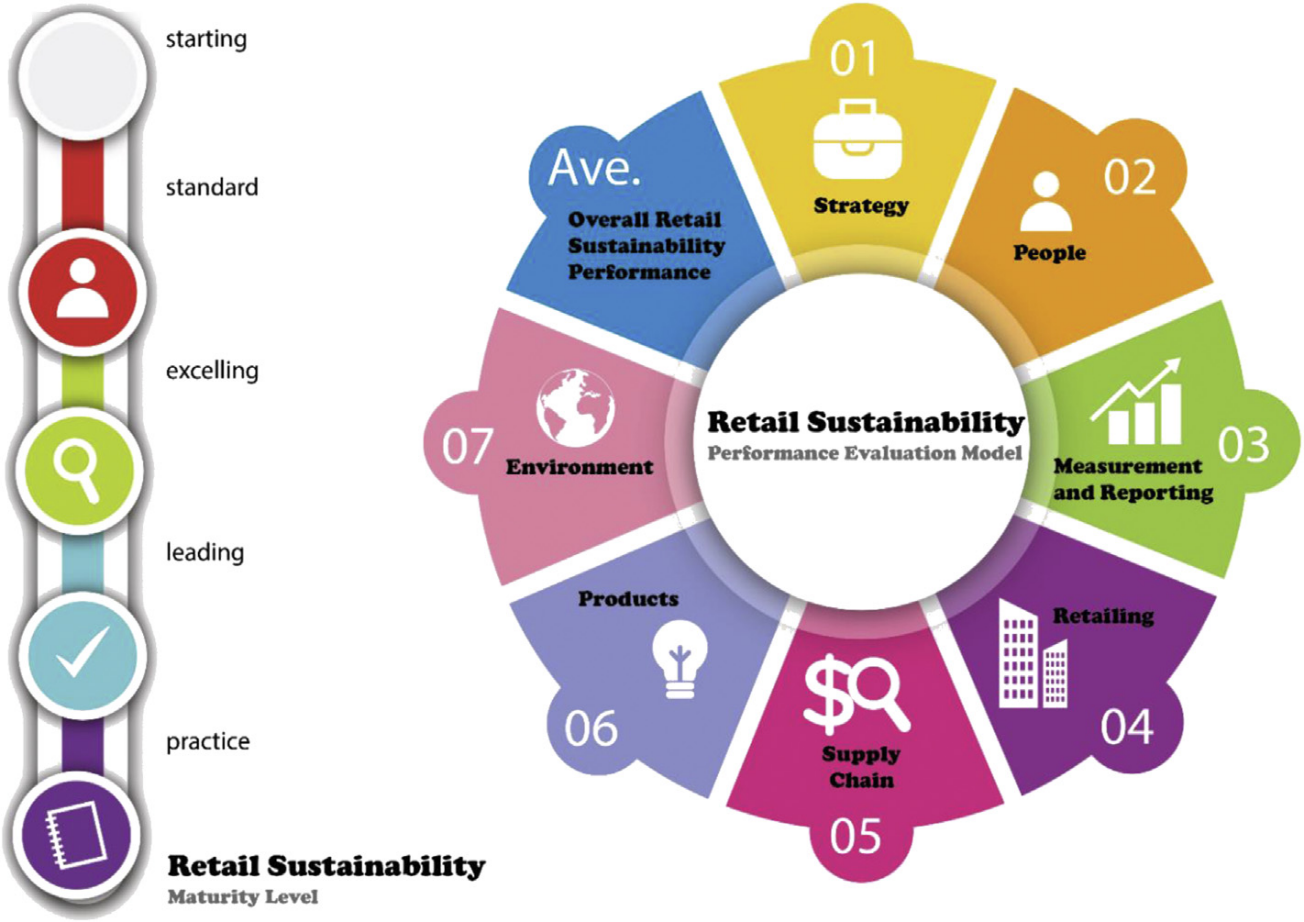
Retail sustainability performance evaluation model (adopted from RILA).

The sustainability performance of retailers is rarely evaluated independently in part due to the significant scale of the organisations, the extent of their horizontal and vertical reach, and the lack of an independent comprehensive framework for retail sustainability performance evaluation. One solution proposed by the Retail Industry Leaders Association (RILA, 2012) is a sustainability management model for performance evaluation. This framework has seven dimensions and 27 criteria that are put together in the following graphic (Figure 1).

The Retail Sustainability Performance Evaluation Model is made up of seven dimensions. These include strategy, people, measurement and reporting, retailing, supply chain, products, and environment. Each dimension is comprised of three to five criteria. In addition, the scale for the evaluation entails five maturity levels of Starting, Standard, Excelling, Leading, and Next Practice. The Dimensions/Sub-dimensions of RILA are presented below in Table 1.

**Table 1:** The dimensions/sub-dimensions of the Retail Industry Leaders Association (RILA).

Coding	Dimensions/Sub-dimensions
**A**	**Strategy**
A.1	Strategy
A.2	Materiality/Risk Identification
A.3	Goals
A.4	Governance & Executive Engagement
A.5	Incentives
**B**	**People**
B.1	Stakeholder Engagement
B.2	Employee Engagement
B.3	Funding Mechanisms
B.4	Business Innovation Mechanisms
**C**	**Measurement and Reporting**
C.1	Metrics & Measurement
C.2	Reporting & Communicating
C.3	Point-of-Purchase Consumer Education
C.4	Marketing Campaigns
C.5	Collaborative Involvement
**D**	**Retailing**
D.1	Stores/Corporate Offices
D.2	Warehouses/DCs
D.3	Data Center & Applications
**E**	**Supply Chain**
E.1	Transportation/Logistics
E.2	Supplier Engagement
E.3	Supply Chain Transparency & Traceability
**F**	**Products**
F.1	Product & Packaging Design and Development
F.2	Owned Manufacturing/Production
F.3	Product & Packaging End-of-Life Stewardship
**G**	**Environment**
G.1	Energy & GHG Emissions
G.2	Water & Wastewater
G.3	Waste & Recycling
G.4	Chemicals & Toxics

#### 2.2.2 The modified RILA sustainability performance evaluation model

There are a number of weaknesses with the RILA's sustainability management and reporting model. One weakness is that it is a self-reporting model. Relying exclusively on self-reporting puts to question the veracity of the reporting and the balance of issues. A second matter of concern is that the RILA model was developed primarily for US retailers and requires adjustment for global application. Some adjustment was made and integrated into a Modified RILA Sustainability Management Model. For instance, the US-based frames of reference for supply chain performance frameworks such as LEED and EPA SmartWay Carrier standards were modified to International ones to allow international comparison. These changes, however, were inadequate on both of the same criteria, namely, self-reporting and US focused.

Accordingly, a new stronger model was developed beyond the Modified RILA. The modification created a four-dimensional model, specifically, the Social, Economic, Environmental, Supply Chain (SEES) model. SEES model was developed using mapping method to identify the most frequent themes in retail sustainability reporting. The increasing popularity of measuring social, environmental, among other CSR and sustainability performance evaluation in tandem with the value added of inter-industry comparability using the economic (and governance), social, environmental, and supply chain in retail industry dimensions therefore have become more appropriate. Additionally, cross-industry and longitudinal comparisons, which were absent the previous RILA model, were introduced in the Modified RILA in order to quantify retail sustainability performance dimensions and criteria. This modification enables independent cross-company comparison and benchmarking as well as multi-year industry sustainability performance evaluation.

#### 2.2.3 The social, economic, environmental, supply chain (SEES) model

As noted, we have taken the Modified RILA Sustainability Management Model and further worked with it modifying the measures/indicators using expert panel independent evaluation. The modification resulted in a four-dimensional model. SEES' four dimensions Economic (EC), social (SO), environmental (EN), and supply chain (SC) constitute the core of sustainability strategy among global retailers. The SEES model evaluates the sustainability performance of retailers based on the economic, social, environmental measures of the Modified RILA Sustainability Management Model and adds supply chain.

SEES was developed using an issue mapping method to identify the most frequent themes in retail sustainability reporting. The modified indicators have proven to be reliable sustainability measurement apparatuses often used in corporate performance evaluation in recent years (Rahdari, 2016; Braendle and Rahdari, 2016). The SEES model strengthens implementation of corporate level strategy by providing quantitative indicators in each performance dimension and allows management to identify strengths and weaknesses of the overall sustainability performance of the company and monitor performance in these dimensions. It also enables cross-country and cross-company comparisons which can inform the decisions of policy makers, business associations, the leaders of the industry, and other stakeholders.

## 3 Methods

### 3.1 Sampling and selection of retailers

To address our research questions, the top 250 Global Powers of Retailing list of 2017 by Deloitte was utilized for the sampling process at the onset. Three main criteria were put into place to filter through the retailers on the list to develop a list of global retailers suitable for the study. The criteria are: a) revenue above $20 billion, b) general retailer, and c) have recently (within the 3 years, 2014–2016) issued a sustainability report in English, German, and French.^1^ Having examined the top 250 retailers using the three-criterion filter, 23 retailers from nine countries were selected for the analyses shown below in Table 2. The sample included the top 10 retailers, except Amazon, where we could not find data. With our sample we were able to cover more than 30% of the Top 250's total retail revenue (Deloitte Touche Tohmatsu, 2017).

**Table 2:** Final set of global retailers.

	Retailer	Country
1	Wal-Mart Stores, Inc.	US
2	Costco Wholesale Corporation	US
3	The Kroger Co.	US
4	Tesco PLC	UK
5	J. Sainsbury plc	UK
6	Lotte Shopping Co., Ltd.	South Korea
7	Carrefour S.A.	France
8	Auchan Holding SA	France
9	Casino Guichard-Perrachon S.A.	France
10	Wesfarmers Limited	Australia
11	Woolworths Limited	Australia
12	Lidl (Schwarz UT KG)	Australia
13	Aldi	Germany
14	Metro	Germany
15	Edeka	Germany
16	Wm Morrison	UK
17	Migros	Switzerland
18	Coop	Switzerland
19	Target Corporation	US
20	Aeon Co., Ltd.	Japan
21	Lowe's Companies, Inc.	US
22	Seven & i Holdings Co., Ltd.	Japan
23	Ahold Delhaize	The Netherlands

The retailers' sustainability reports and websites were used as the main sources for data collection. The companies' recent (up to 3 years, 2014–2016) sustainability reports were analyzed according to criteria set above. The reports were read in detail in one of the three languages of English, German, and French. Year 2014 was selected as the starting year because it was in 2014 that the Rana Plaza industrial disaster occurred. In that event, a building collapse resulted in the death of over 1100 workers and more than 2000 severe injuries in Bangladesh. In the aftermath, large global retailers declared an intention to work collectively to improve working conditions and subsequently formed a buyer's alliance to monitor and improve their supply chain partners in terms of sustainability performance generally. Partnering with the International Labor Organisation (ILO), this initiative creates an opportunity for multiple stakeholders to work together with leading retail firms in global value chains and a related opportunity for researchers to investigate this important area of practice and reform.

Overall Scores (Dimension-level) – To measure sustainability, the three traditional component areas of environmental, social and economic sustainability (Triple Bottom Line) originally described by Elkington (1997), have been adopted by the 23 retailers and form the basis for this study. Not surprisingly, the focus of retailers to date has been on economic and social issues, as it appears that retailers believe that addressing environmental sustainability would compromise the economic bottom line (Jones et al., 2011a, b).

The model's 27 items used to understand overall sustainability performance were grouped into four broader categories and divided as follows: economic (9 items), social (5 items), supply chain (6) and environmental (7 items) all within the SEES model. To measure the sustainability performance scorecard, a seven-dimensional RILA framework that included 27 criteria/items was also used. The scoring scale had five levels: Starting, Standard, Excelling, Leading, and Next Practice and definitions for each of these levels was provided for each item. For instance, the Stakeholder Engagement sub-dimension from People & Tools dimension had the following scale (Table 3).

**Table 3:** Sample scale of each sub-dimension.

People & Tools					
Stakeholder Engagement	Starting 0-20	Standard 21-40	Excelling 41-60	Leading 61-80	Next Practice 81-100
	Identifies key stakeholders Identifies some stakeholder concerns on a periodic basis but no defined method of proactive engagement	Assesses stakeholder concerns systematically through materiality analysis Establishes and communicates methods of stakeholder engagement	Addresses stakeholders concerns through materiality analysis and identifies some KPIs from the process Builds relationships with key stakeholders	Identifies comprehensive list of KPIs through stakeholder engagement process Establishes and communicates methods of stakeholder engagement by type and stakeholder group, including frequency of engagement Incorporates feedback from key stakeholders into sustainability strategy	Consistently monitors and reports publicly on KPIs identified through stakeholder engagement

The scoring ranged from 0-100 (in increments of 20) based on the level/scale that matched the performance of each firm in each sub-dimension/item. The average score for each (sub-) dimension is the average of the scores of items in each (sub-) dimension for all firms. The average score for each firm is the average of the scores of items in each (sub-) dimension for that firm.

## 4 Results and discussions

### 4.1 Results: sustainability priorities and CSR performance in retail industry (RQ 1)

The results discussed are based on disclosures from sustainability reports of the retailers which demonstrate the level of adoption of sustainability practices. We will discuss how the different retailers studied devise their sustainability strategy to include economic, social, environmental, and supply chain dimensions as well as the value-action gap in their sustainability strategy. Key factors have emerged from the examination of the sustainable strategic priorities adopted by retailers across the globe. These factors can serve as a checklist and as a starting point for retailers in managing their sustainability. The discussion which follows is divided into two subsections: the first dealing with the dimension level and second with the criteria level.

#### 4.1.1 Dimension level

**Figure 2:**
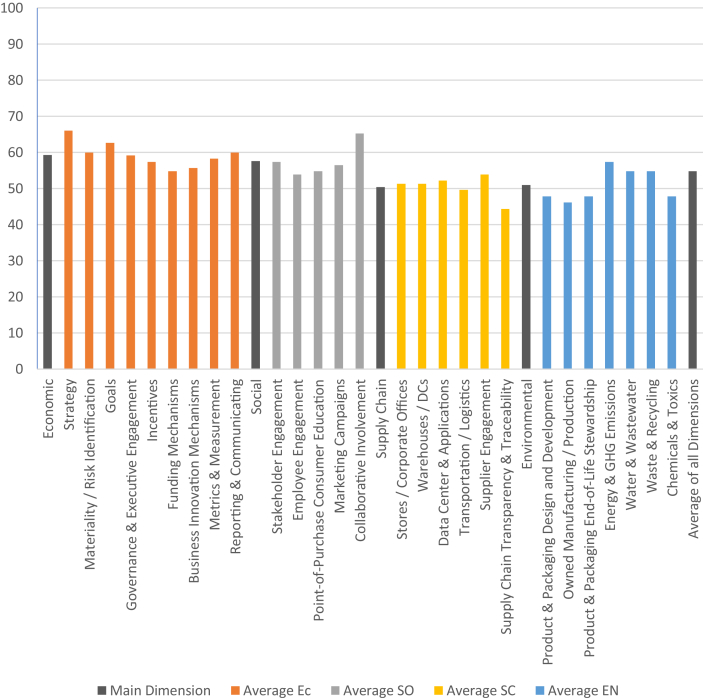
Overall scores (dimension-level).

Figure 2 - Overall Scores presents the performance scores in all 27 dimensions for the 23 retailers analyzed. The retailers score highest in the economic dimension (59.3/100), followed by the social dimension (57.57), environmental (50.93) as well as supply chain (50.43). Within the dimensions, strategy (or the question how much sustainability strategy aligns across departments and with overall corporate strategy) scored highest with an average of 66.09. At the other end of the scale, Supply Chain Transparency & Traceability scored lowest with an average of 44.35. The latter finding is concerning as the research show that the shareholder wealth impacts of supply chain disruptions are significantly more negative and typically greater in magnitude than in many other types of operational, marketing, and financial events (Hendricks and Singhal, 2003). The findings suggest that global retailers have a relatively balanced view of different sustainability dimensions but could benefit from greater attention to supply chains.

##### 4.1.1.1 Economic (EC) dimension

**Figure 3:**
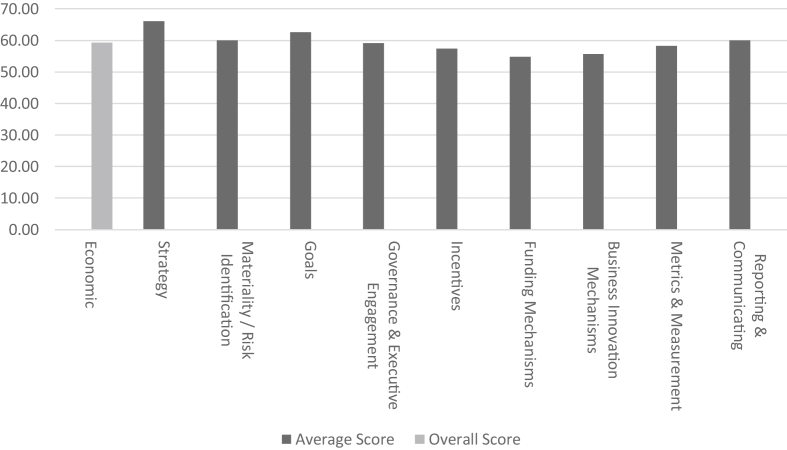
Economic (EC) dimension.

Amongst retailers, there seems to be a focus on the economic dimension (average score 59.3), likely best explained by the dominant neoclassical economics view that the social responsibility of businesses is to maximize profits and that firms “can do good – but only at their own expense” (Friedman, 1970, p. 4). In other words, the main responsibility from a finance point of view is to maximize the profits of shareholders (Reinhardt et al., 2008) although this is not the legal requirement (Sheehy and Feaver, 2014). Within the economic dimension, Figure 3, (overall average score 59.3), we can see the different elements ranging between Strategy (66.09) and Funding Mechanisms (54.78), i.e. funding dedicated for sustainability programs.

##### 4.1.1.2 Social (SO) dimension

In 2009, the EC developed sustainable policies and recognized that, “The retail sector has undoubtedly a key role to play in sustainable production and consumption.” This key role is based upon its critical position of retail in the consumption chain, a position which enables it to influence both production and consumption, and possibly, where retailers get involved in accepting return of used products, in the product lifecycle (Kotzab et al., 2011).

**Figure 4:**
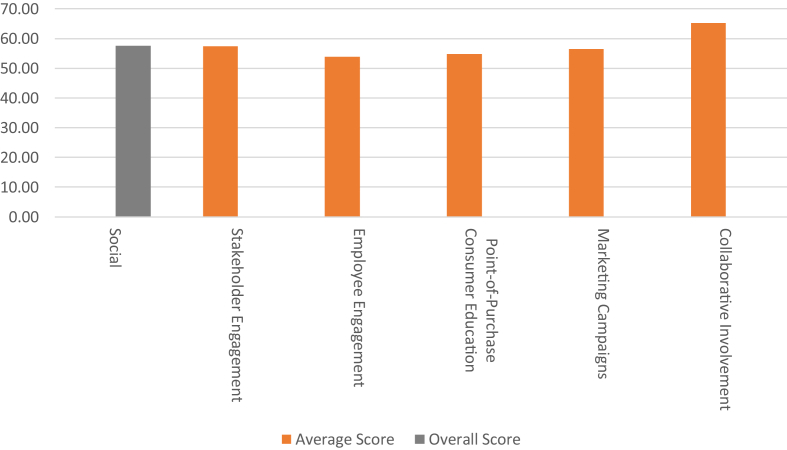
Social (SO) dimension.

In terms of the social dimensions, as seen in Figure 4, on average retailers scored high on collaborative involvement (65.22). It would seem that there is a trend towards actively sharing sustainability information with peers. On the other side of the dimension, retailers could do more in terms of employee engagement (53.91). The results are interesting, as engaging employees would seem to be crucial success factor for the retail sector.

##### 4.1.1.3 Supply chain (SC) dimension

**Figure 5:**
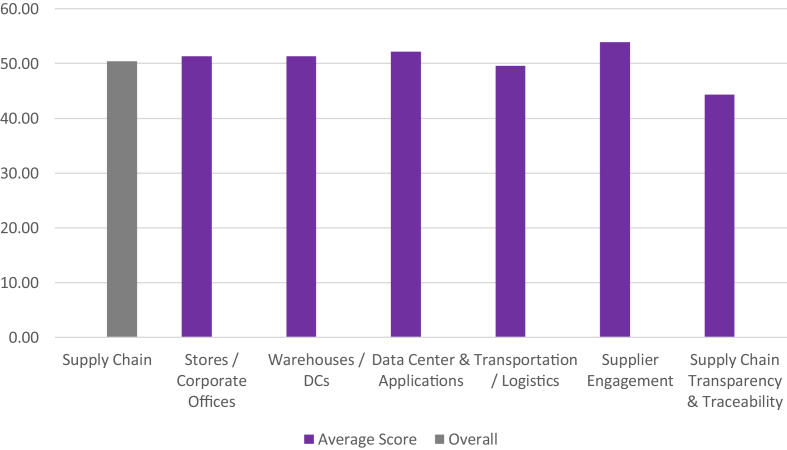
Supply chain (SC) dimension.

CSR in supply chain management (SCM) has gained increased attention in terms of research recently. Studies have discussed stakeholder interests, performance evaluation, ethical sourcing, and sustainable production (Feng et al., 2017). In regard to the retailers, the overall average score of 50.43 for the Supply Chain dimension in Figure 5, was the lowest amongst the measured dimensions. Especially in the area of supply chain transparency and traceability the average score of 44.35 was the lowest amongst the measured 27 criteria. This finding should initiate a process within retailers to improve sustainability in supply chains, particularly as research confirms that shareholder wealth is impacted in significantly negative and typically greater in magnitudes by supply chain disruptions than many other types of operational, marketing, and financial events (Hendricks and Singhal, 2003).

##### 4.1.1.4 Environmental (EN) dimension

Since the 1960s, diverse stakeholders, including the government, employees, the media, and the public, have become increasingly concerned with organizations' commitment to governance standards, environmental issues, social investment, and community involvement. In 1983, the United Nations convened the World Commission on Environment and Development, also known as Brundtland Commission, to address widespread concern about growing socioeconomic inequalities, the depletion of natural resources, and environmental destruction (United Nations Global Compact, 2017). The whole discussion that developed into the triple bottom line was initially primarily understood as environmental sustainability (Crane and Matten, 2007).

**Figure 6:**
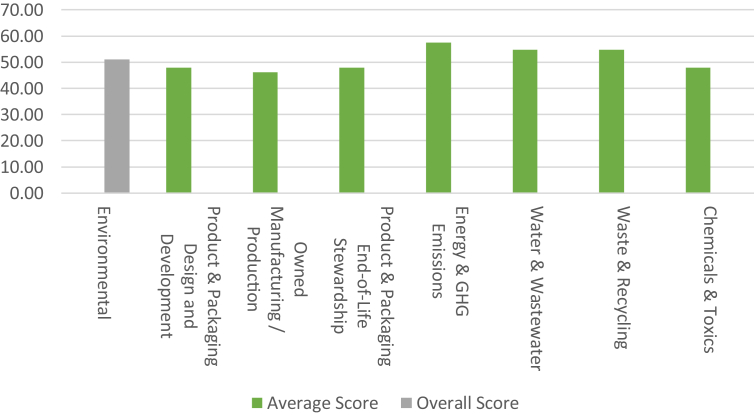
Environmental (EN) dimension.

As shown in Figure 6, although environmental sustainability was critical to starting the larger global sustainability dialogue, environmental performance in our study – with an average score of 50.93 – did not reflect that priority nor its importance. Retailers do not seem to focus on environmental issues in their activities or their reporting. There may be a variety of reasons for this including that the retailers themselves may not consider their operations as being as heavily dependent upon environmental resources as manufacturing or primary resource businesses, and so might not see themselves as having a very large environmental impact compared to those other industries.

We turn next to examine data at the criteria level of the model. We examine the social, supply chain and environmental perceptions of retailers.

#### 4.1.2 Criteria level

In this subsection, we review the criteria level results of our model. As with the prior subsection, we provide a figure with brief commentary.

##### 4.1.2.1 Social criteria

**Figure 7:**
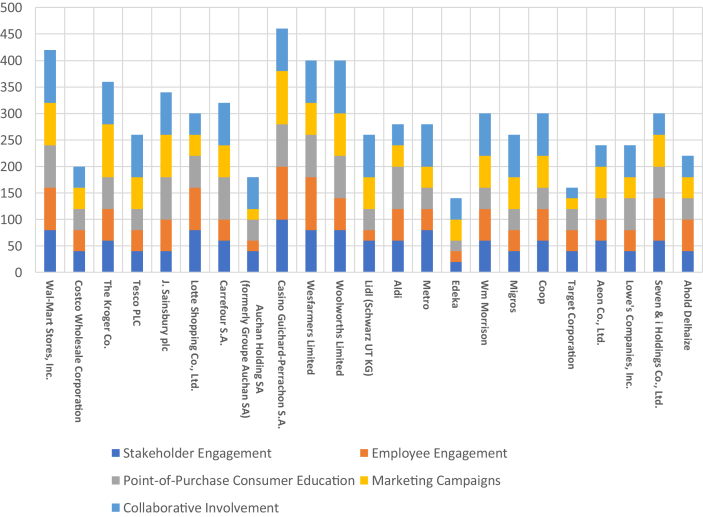
Retailer's perception of performance in social dimension.

At the criteria level, the social dimension is composed of the following: stakeholder engagement, point-of-purchase consumer education, collaborative involvement, employee engagement and marketing campaigns (see Figure 7).

##### 4.1.2.2 Supply chain (criteria-level)

**Figure 8:**
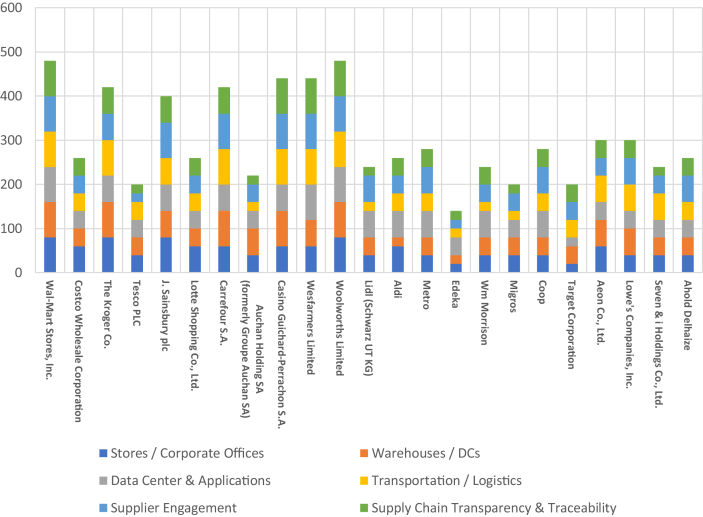
Retailer's perception of performance in supply chain dimension.

The Supply Chain criteria is composed of the following: stores/corporate, data center and applications, supplier engagement, warehouses/DCs (distribution centers), transportation/logistics, and supply chain transparency and traceability. In the supply chain dimension, none of the retailers were able to reach the Best Practice level in any of the criteria. This is, as discussed above particularly worrying, as supply chain disruptions can negatively impact retailers in pursuing their corporate-wide global strategic priorities (Figure 8).

##### 4.1.2.3 Environmental (criteria-level)

**Figure 9:**
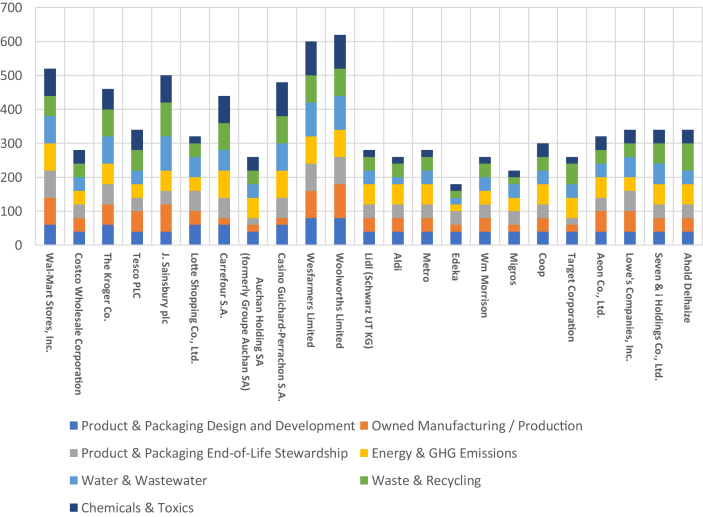
Retailer's perception of performance in environmental dimension.

The environmental criteria are: product and packaging design and development, product and packaging end-of-life stewardship, water and wastewater, chemicals and toxics, owned manufacturing/production, energy and GHG emissions, waste and recycling (Figure 9) In terms of the environmental dimension it seems that retailers have started coordinating energy efficiency policies across their operations and the value chain. Having said that, and as much as the whole discussion around the triple bottom line has initially been understood as environmental sustainability, there is still a lot of room for retailers to address the environmental issues.

The results of the all retailer's sustainability performance based on the modified RILA model and under SEES model is presented in the Table A, Table B in Appendix.

### 4.2 (RQ 2) how does the CSR performance of global retailers compare across three regional markets?

Analyzing the performance of global retailers using the modified SEES brings to light differences among major retail markets. In this section we analyse and evaluate the US, Europe and Australasia.

**Figure 10:**
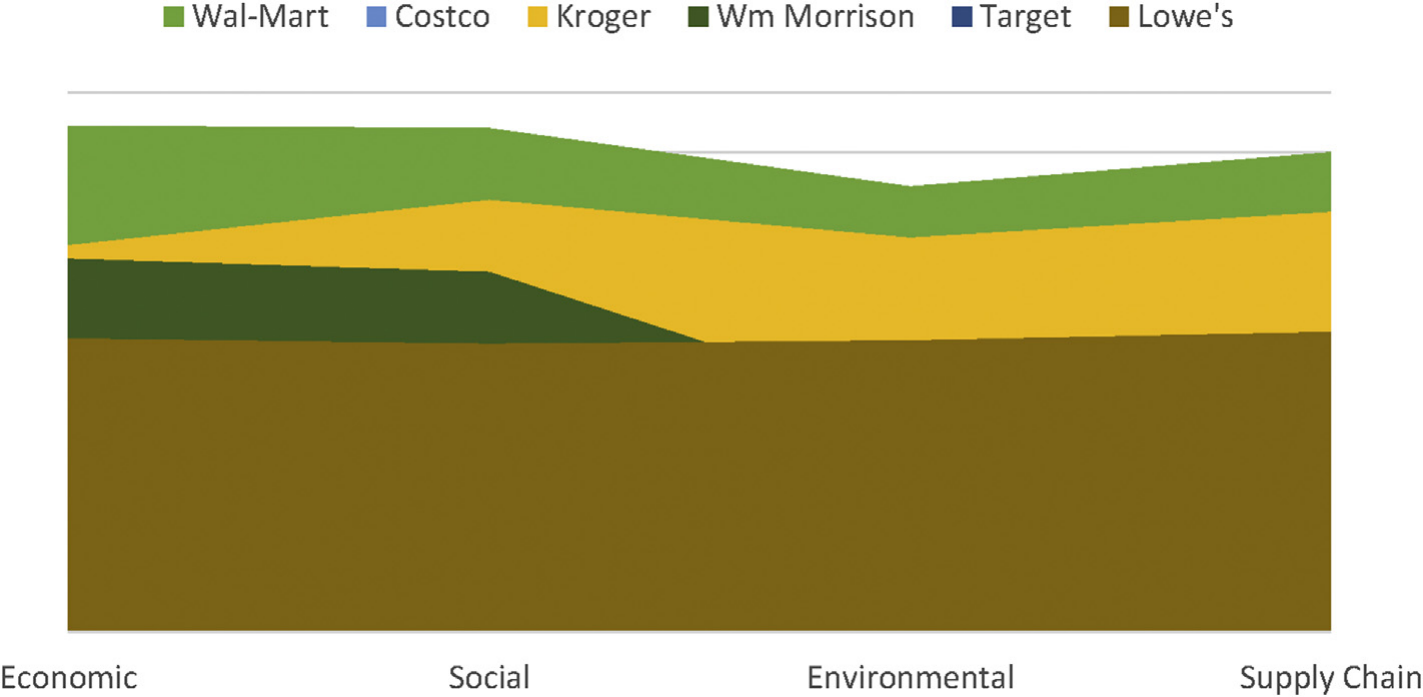
US retail leaders - SEES analysis model.

**Figure 11:**
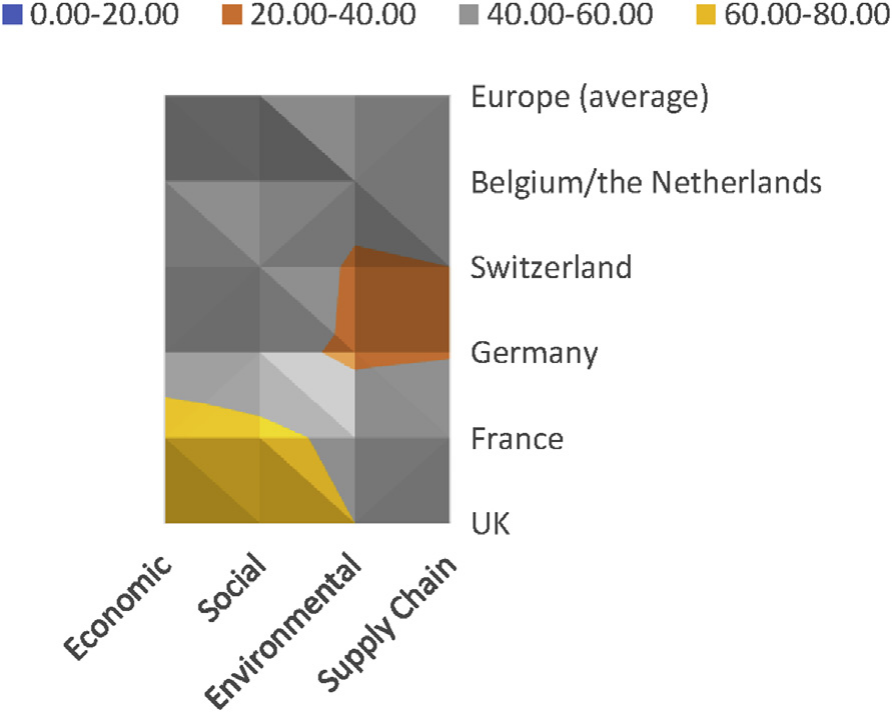
European retail leaders - SEES analysis model.

**Figure 12:**
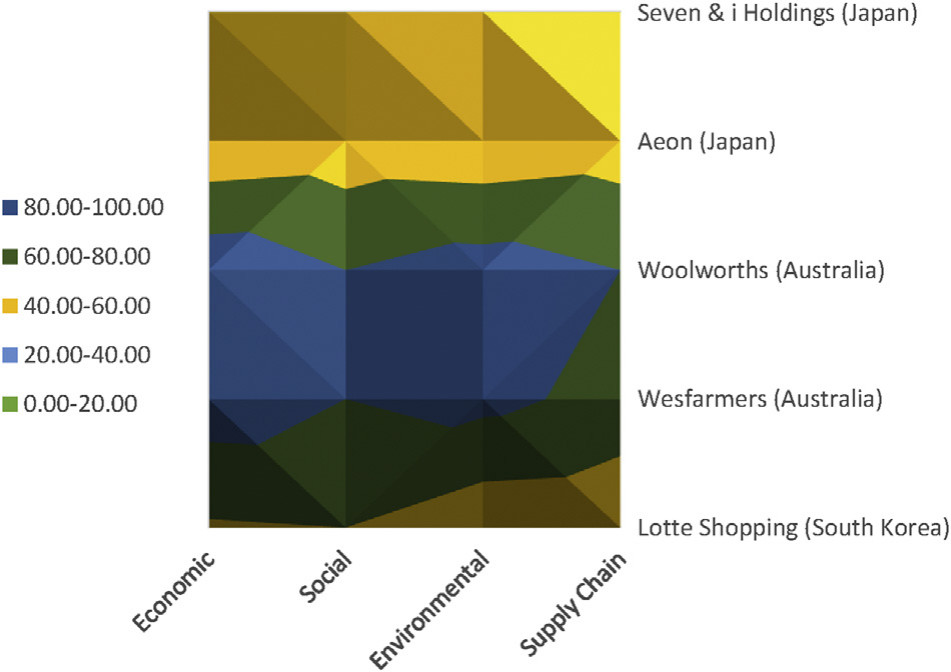
Australasian retail leaders - SEES analysis model.

In Europe, on average, the French (62/08%) and the British retailers (57/50%) are in the lead perhaps reflecting better legislative support and strong track record of voluntary initiatives. A region-based comparison demonstrates that US retailers (overall score 54/07%) on average are ahead of European retailers (50/81%) by a small margin in all four dimensions. It is evident from the Figure 10, the US retail leaders are bunched lower on the supply chain sustainability performance score, with the notable exceptions of Walmart and Kroger. Further, it also provides evidence of a stronger interest in sustainable supply chains in Europe (Figure 11). Finally, as Figure 12 indicates, in the Australasian region, the retailers are divisible as Australian and Asian.

A distinct line of research studies focuses on sustainable supply chain management practice and its linkage to organizational performance (Seuring and Gold, 2013). The literature in this field has been inconclusive. For example, while some studies have found a direct and positive relationship between sustainable supply chain management practices and organizational performance (Zhu et al., 2005), other studies have reported a negative relationship. Yet other studies have drawn attention to the necessity of considering moderating and mediating variables in understanding the relationships (Zhu et al., 2012). One significant weakness of many of these studies is their reliance on surveys to examine managerial perceptions of organizational performance instead of more concrete and objective organizational performance data. While such data are available from publicly available sources such as firms' website disclosures relating to sustainability reporting, CSR reports, more general annual reports (e.g. Zhu et al., 2012), the prior research does not appear to engage with it as much is it could.

In sum, although there has been research on a variety of sustainable supply chain issues, they have not been holistic, data driven or facilitated global comparisons or evaluation. One model that allows such work, the Social Economic Environmental Supply Chain (SEES) model is designed to address these important issues. Benchmarking of sustainable supply chain performance is important as benchmarking allows enables policymakers, researchers and managers to have a complete overview of the performance and progress of their sustainable supply chains across the various dimensions. Most importantly, it allows both the public and the organs of global sustainability to monitor and evaluate performance in this major global industry.

## 5 Concluding remarks

This study examined and explored the CSR strategic priorities and practices of leading global retailers with particular attention to their supply chains. Further, it evaluated the state of CSR strategy in the retail industry using a new analytical tool. The key findings of the study are that global retailers have a relatively balanced approach to the management of the different dimensions of CSR including supply chains in general. The data indicates, however, that European retailers outperform their American counterparts placing a higher priority on supply chain sustainability performance. Further, it demonstrates a weaker approach to the environment among American retailers and a stronger approach to the social dimension among European retailers. Finally, the study shows that Australasian retailers perform at a level which places them between the European and American retailers.

The study offers some academic and practical contributions. From an academic perspective the study contributes to the literature in the following ways. First, previous studies on CSR and sustainability performance in retail industries were limited to a one or few retail firms. Further, these studies were single country focused (see Wang and Sarkis, 2013; Walker and Jones, 2012; Zhu et al., 2005; Islam and Deegan, 2010). While these studies advanced understanding about these retailers' CSR and sustainability performance, they had both a limited focus and reach. By way of contrast, the current study has focused exclusively on global retailers' CSR performance and broad regional markets. Accordingly, the current study enables a broader and more holistic understanding of CSR performance of global retailers including their supply chain partners. Further, it offers comparative opportunities across different regions. This study is the first to examine international global retailers attending to their different regional focuses and to provide an account of their CSR and sustainability. Having said that, our sample of 23 retails after applying three filter on the Top 250 global retailers can certainly be a limitation in itself.

One significant limitation of previous studies is their reliance on surveys for examining CSR and supply chain performance (e.g. Zhu et al., 2012). More objective data sourced from annual reports and other publicly available sources such as firms' CSR reports, has been used in the current study and so allowed the current study to offer a significantly more reliable evaluation.

Finally, the study provides a new analytical tool as an improvement on the existing models. The new framework, the Social Economic Environmental Supply Chain (SEES) model, is designed to address important issues to allow policymakers, researchers and managers to have better insight into the performance and progress of their sustainable supply chains across the various dimensions.

The findings of the study have practical value as well for the retail industry and others. Firstly, the study highlights the important role of retailers in contributing to new sustainability norms in business and to achieving the SDGs. The paper shed further light on current CSR practices among leading global retailers. Decision-makers in the large retailers are now better positioned to understand which performance dimension they need to improve, and able to identify and evaluate their own positions in comparison with other regional and global competitors. Secondly, the study identified the CSR dimensions of each of the leading global retailers' strategy. It further identified which criteria contributed most to each retailers' CSR performance. These analyses were supported by the SEES model which offered new insights into the four dimensions of CSR in retail and how global retailers are performing on each of those dimensions. Regulators and other policymakers can investigate retailer's performance and use the findings of their investigations for their policy initiatives or decisions.

Thirdly, the study demonstrated how the modified RILA and SEES Analysis Model can used to evaluate retail sustainability performance and to score retailers' sustainability performance. This powerful model is readily used as a steppingstone for conducting further independent longitudinal sustainability performance studies and cross-company comparison and benchmarking. Performance benchmarking across different regions allows policymakers, researchers and managers to have a complete overview of the performance and progress of their sustainable supply chains across the various dimensions. Finally, the findings suggest that, by and large, CSR is making its way throughout the retail supply chain. The study also makes it clear, however, that there is insufficient uptake to support the theory that businesses can be left to their own devices to address sustainability. In the absence of public regulation, there is insufficient attention and pressure to significantly improve performance. Accordingly, some form of increased public regulation is necessary to achieve SDG goals. The SEES model allows for serious evidence based critique of the behavior of firms.

The limitations of our study are clear. As described above, the data is drawn from a selected group of the largest global retailers and over the limited time period of three years. Conclusions from the study cannot be generalized to medium or smaller sized firms and indeed, may not be applicable even to other large firms, particularly to those outside the retail sector. Further, given the time frame for this work it is uncertain to what extent practices may extend beyond the study period.

Of particular importance are the dramatic changes the COVID-19 pandemic has imposed on the economy including retailers and consumers. COVID has had an overwhelming impact on retailers, negatively on bricks and mortar businesses but markedly positively on those operating online. How these different businesses will deal with sustainability in their businesses and supply chains is fruitless speculation at this point. To this end, however, the current study provides a benchmark for future studies of global retailers CSR practices allowing comparison between them just prior to the onset of the pandemic. Further, global retailers will need to consider how they are able to secure their supply chains particularly where suppliers are in emerging economies and potentially unable to fulfill their contractual obligations due to illness. In addition, retailers will have to be able to address their own financial sustainability, which may be at risk, any trade-offs they may need to make. All of these topics are certainly worthy of future investigation.

A final limitation of our study is that we have relied exclusively on the self-published reports of the retailers. Although many of the reports were assured by third parties, not all were across the time period. The potential for self-serving bias in the data collection and reporting is significant and suggests a cautious approach to this aspect of the current study. To remedy this limitation, other studies may consider other approaches to data collection to work with independently verifiable information.

## Declarations

### Author contribution statement

U. Braendle, B. Sheehy, H.Z. Khan and G. Rexhepi: Conceived and designed the experiments; Performed the experiments; Contributed reagents, materials, analysis tools or data; Wrote the paper.

Rahdari: Conceived and designed the experiments; Analyzed and interpreted the data; Wrote the paper.

S. Sepasi: Conceived and designed the experiments; Analyzed and interpreted the data; Wrote the paper.

### Funding statement

This research did not receive any specific grant from funding agencies in the public, commercial, or not-for-profit sectors.

### Competing interest statement

The authors declare no conflict of interest.

### Additional information

No additional information is available for this paper.

## Figures and Tables

**Table A:** Retail Sustainability Performance Evaluation Scorecard under the modified Retail Industry Leaders Association (RILA) model

Num.	Code	Dimension - Criteria	Average Score
	**A**	**Strategy**	**61/04**
1	A.1	Strategy	66/09
2	A.2	Materiality/Risk Identification	60/00
3	A.3	Goals	62/61
4	A.4	Governance & Executive Engagement	59/13
5	A.5	Incentives	57/39
	**B**	**People**	**55/43**
6	B.1	Stakeholder Engagement	57/39
7	B.2	Employee Engagement	53/91
8	B.3	Funding Mechanisms	54/78
9	B.4	Business Innovation Mechanisms	55/65
	**C**	**Measurement and Reporting**	**58/96**
10	C.1	Metrics & Measurement	58/26
11	C.2	Reporting & Communicating	60/00
12	C.3	Point-of-Purchase Consumer Education	54/78
13	C.4	Marketing Campaigns	56/52
14	C.5	Collaborative Involvement	65/22
	**D**	**Retailing**	**51/59**
15	D.1	Stores/Corporate Offices	51/30
16	D.2	Warehouses/DCs	51/30
17	D.3	Data Center & Applications	52/17
	**E**	**Supply Chain**	**49/28**
18	E.1	Transportation/Logistics	49/57
19	E.2	Supplier Engagement	53/91
20	E.3	Supply Chain Transparency & Traceability	44/35
	**F**	**Products**	**47/25**
21	F.1	Product & Packaging Design and Development	47/83
22	F.2	Owned Manufacturing/Production	46/09
23	F.3	Product & Packaging End-of-Life Stewardship	47/83
	**G**	**Environment**	**53/70**
24	G.1	Energy & GHG Emissions	57/39
25	G.2	Water & Wastewater	54/78
26	G.3	Waste & Recycling	54/78
27	G.4	Chemicals & Toxics	47/83
**Average Sustainability Performance of Global Retailers under**			**54/85**

**Table B:** Retail Sustainability Performance Analysis under the Social, Economic Environmental, Supply Chain (SEES) Model

Num.	New Code	Dimension - Criteria	Average
	**EC**	**Economic**	**59/32**
1	EC1	Strategy	66/09
2	EC2	Materiality/Risk Identification	60/00
3	EC3	Goals	62/61
4	EC4	Governance & Executive Engagement	59/13
5	EC5	Incentives	57/39
6	EC6	Funding Mechanisms	54/78
7	EC7	Business Innovation Mechanisms	55/65
8	EC8	Metrics & Measurement	58/26
9	EC9	Reporting & Communicating	60/00
	**SO**	**Social**	**57/57**
10	SO1	Stakeholder Engagement	57/39
11	SO2	Employee Engagement	53/91
12	SO3	Point-of-Purchase Consumer Education	54/78
13	SO4	Marketing Campaigns	56/52
14	SO5	Collaborative Involvement	65/22
	**SC**	**Supply Chain**	**50/43**
15	SC1	Stores/Corporate Offices	51/30
16	SC2	Warehouses/DCs	51/30
17	SC3	Data Center & Applications	52/17
18	SC4	Transportation/Logistics	49/57
19	SC5	Supplier Engagement	53/91
20	SC6	Supply Chain Transparency & Traceability	44/35
	**EN**	**Environmental**	**50/93**
21	EN1	Product & Packaging Design and Development	47/83
22	EN2	Owned Manufacturing/Production	46/09
23	EN3	Product & Packaging End-of-Life Stewardship	47/83
24	EN4	Energy & GHG Emissions	57/39
25	EN5	Water & Wastewater	54/78
26	EN6	Waste & Recycling	54/78
27	EN7	Chemicals & Toxics	47/83
**Average Sustainability Performance of Global Retailers**			**54/85**

## Footnotes

1 Two co-authors are fluent in German and French and assisted the team in evaluating the German and French language primary sources.
